# Quantitative susceptibility mapping using plug-and-play alternating direction method of multipliers

**DOI:** 10.1038/s41598-022-22778-w

**Published:** 2022-12-15

**Authors:** Srikant Kamesh Iyer, Brianna F. Moon, Nicholas Josselyn, Robert M. Kurtz, Jae W. Song, Jeffrey B. Ware, S. Ali Nabavizadeh, Walter R. Witschey

**Affiliations:** 1 Department of Radiology, University of Pennsylvania, Philadelphia, PA USA; 2 Department of Bioengineering, University of Pennsylvania, Philadelphia, PA USA; 3 Perelman Center for Advanced Medicine, South Pavilion, Rm 11-155, Philadelphia, PA USA

**Keywords:** Biotechnology, Biomarkers, Medical research

## Abstract

Quantitative susceptibility mapping employs regularization to reduce artifacts, yet many recent denoisers are unavailable for reconstruction. We developed a plug-and-play approach to QSM reconstruction (PnP QSM) and show its flexibility using several patch-based denoisers. We developed PnP QSM using alternating direction method of multiplier framework and applied collaborative filtering denoisers. We apply the technique to the 2016 QSM Challenge and in 10 glioblastoma multiforme datasets. We compared its performance with four published QSM techniques and a multi-orientation QSM method. We analyzed magnetic susceptibility accuracy using brain region-of-interest measurements, and image quality using global error metrics. Reconstructions on glioblastoma data were analyzed using ranked and semiquantitative image grading by three neuroradiologist observers to assess image quality (IQ) and sharpness (IS). PnP-BM4D QSM showed good correlation (β = 0.84, R^2^ = 0.98, *p* < 0.05) with COSMOS and no significant bias (bias = 0.007 ± 0.012). PnP-BM4D QSM achieved excellent quality when assessed using structural similarity index metric (SSIM = 0.860), high frequency error norm (HFEN = 58.5), cross correlation (CC = 0.804), and mutual information (MI = 0.475) and also maintained good conspicuity of fine features. In glioblastoma datasets, PnP-BM4D QSM showed higher performance (IQ_Grade_ = 2.4 ± 0.4, IS_Grade_ = 2.7 ± 0.3, IQ_Rank_ = 3.7 ± 0.3, IS_Rank_ = 3.9 ± 0.3) compared to MEDI (IQ_Grade_ = 2.1 ± 0.5, IS_Grade_ = 2.1 ± 0.6, IQ_Rank_ = 2.4 ± 0.6, IS_Rank_ = 2.9 ± 0.2) and FANSI-TGV (IQ_Grade_ = 2.2 ± 0.6, IS_Grade_ = 2.1 ± 0.6, IQ_Rank_ = 2.7 ± 0.3, IS_Rank_ = 2.2 ± 0.2). We illustrated the modularity of PnP QSM by interchanging two additional patch-based denoisers. PnP QSM reconstruction was feasible, and its flexibility was shown using several patch-based denoisers. This technique may allow rapid prototyping and validation of new denoisers for QSM reconstruction for an array of useful clinical applications.

## Introduction

Quantitative susceptibility mapping (QSM) is a magnetic resonance imaging technique that maps the spatial distribution of tissue magnetic susceptibility using information from the MR signal phase^1^. As a non-invasive imaging tool, it can provide detailed information about pathologic tissue biometals^2,3^, and measure blood oxygen saturation^4^. In several clinical applications, QSM was useful for imaging hemorrhagic stroke^5^, impaired tissue oxygen consumption^6^ and neuronal demyelination^7^. Obtaining accurate and precise measurements of magnetic susceptibility using QSM involves finding solutions to a challenging inverse problem. While advancements have been made in the development of techniques for reconstructing susceptibility maps from the measured signal phase, the reconstruction process nevertheless remains a challenging ill-posed inverse problem^8,9^. Moreover, recent studies show that estimated susceptibility maps depend partly on the processing algorithms chosen^9,10^.

A simple direct inversion approach for QSM, truncated k-space division (TKD)^11^, reduces streaking artifacts by using a truncated approximation of the dipole kernel Fourier domain representation. This allows for rapid estimation of susceptibility maps but leads to severe underestimation of susceptibilities and amplifies image noise. An alternative approach to QSM reconstruction involves the use of the compressed sensing (CS) framework^4,9^. CS models for QSM use a combination of least squares data fidelity constraint and a data regularization (sparsifying) constraint for artifact removal. Most popular regularized QSM reconstruction models utilize regularizers such as Thikhonov constraint^12^, total variation (TV)^13,14^ constraint, total generalized variation (TGV)^15^ or their variants^9,12,15^. These types of constraints penalize the gradient of the estimated susceptibility maps. These models may cause artifacts such as oversmoothing, loss of contrast, blurring of edges, and errors due to mismatch between prior and the estimated susceptibility map^9^. Fixed regularizers, which assume data models such as piece-wise-smoothness, do not conform well with the underlying susceptibility map, as they may contain many features and textures that cannot be adequately captured by simplistic fixed sparsity models. For neuroimaging applications, the use of fixed prior models produces images with low error and high region-of-interest (ROI) accuracy^9^ when assessing in large regions such as the putamen and caudate; but often suffer from smoothing of vascular structures and blurring of low contrast regions such as complex folded gray-white matter structures^8,16,17^.

Recent technical developments in image denoising and broadly in the field of image restoration may find use in the QSM reconstruction problem. The non-local means (NL means) filter^18^ (or its equivalent the unsupervised, information-theoretic, adaptive filter (UINTA),^14^) leverages the self-similarity and non-locality property of the image to build a powerful denoising algorithm. The basic principle of NL means is to build a pointwise estimate of the image where each pixel is estimated as a weighted sum of pixels centered at regions showing similarity to the region neighboring the estimated pixel. Another technique based on the extension of the non-local filtering approach, block matching and collaborative filtering technique (BM3D)^19^ is based on the enhanced sparse representation in the transform domain that is achieved by grouping similar 2D patches of the image in a larger 3D array and filtering the coefficients of after the application of a suitable 3D transformation. While these non-local techniques produce high quality images and have been applied to applications such as MRI denoising, their application to inverse problems such as image reconstruction or QSM has been a challenge since most patch-based non-local denoisers are not variational and do not have an explicit regularizer.

Plug-and-play (PnP) priors is a new approach for model-based image reconstruction using denoising^20^. Venkatakrishnan et al.^20^ introduced PnP for image reconstruction by replacing the proximal operator in an iterative algorithm with a non-local patch-based denoiser. The advantage of PnP is its flexibility; any newly developed denoiser can be used without modification of the underlying algorithm. High quality results were shown for applications such as Fourier ptychography^21^ and inverse scattering in electron micrographs^22^ using PnP-alternating direction method of multipliers (ADMM)^23^. In MRI, non-local patch-based approaches were applied to image denoising^24^ and reconstruction of undersampled MR data^25^. There are currently no available techniques for QSM that employ patch-based denoising during reconstruction.

In this work, we sought to develop and apply the PnP-ADMM framework for QSM. We show that PnP-ADMM QSM reconstruction has good performance in the 2016 QSM reconstruction challenge when compared to several published QSM reconstruction approaches and to multi-orientation QSM reconstruction^12,13,15^. To demonstrate the flexibility of PnP-ADMM, we show QSM reconstructions using several state-of-the-art denoisers that do not have explicit regularizers. Finally, we apply PnP-ADMM QSM reconstruction to the application of magnetic susceptibility mapping in patients diagnosed with glioblastoma multiforme (GBM).

## Theory

In materials with isotropic magnetic susceptibility $\chi$, the relationship between susceptibility and the local magnetic field $\phi$ can be formulated as a convolution of the susceptibility distribution with the unit dipole $d$^7,12^

1 $$\phi = d*\chi + n$$

Here $n$ is the estimated noise in the local field and $*$ is the convolution operator. This equation can be expressed in the Fourier domain as

2 $$F\phi = DF\chi + Fn$$

where *D* is the magnetic dipole kernel represented in the Fourier domain, and *F* is the Fourier operator. Assuming that noise *n* is an independent and identically distributed (i.i.d) Gaussian random variable, a statistically optimal least squares solution is given by the constrained formulation

3 $$\left| {\left| {F^{H} DF\chi - \phi } \right|} \right|_{2}^{2} < \sigma^{2} ,$$

Here $F^{H}$ the inverse Fourier operator and $\sigma$ is the noise standard deviation. Since Eq. (3) is severely ill-conditioned, regularized QSM methods use a priori information about the susceptibility map to regularize the solution in a CS framework. This helps to improve conditioning and reduce dipole inversion artifacts. The unconstrained regularized QSM formulation^12^ is given by

4 $$\frac{\mu }{2}\left| {\left| {F^{H} DF\chi - \phi } \right|} \right|_{2}^{2} + \alpha \varphi \left( \chi \right)$$

The Thikhonov constraint^12^, TV^12^, and TGV^15^ are commonly selected for the constraint $\varphi \left( \chi \right).$ $\alpha$ is a tunable weight that controls that amount of regularization applied. For example, a TV based implementation based on a variant of Eq. (4) is of the form^12,26^

5 $$\frac{1}{2}||M(F^{H} DF\chi - \phi )||_{2}^{2} + \alpha \left| {\left| {W\nabla \chi } \right|} \right|_{1}^{{}}$$

Here $\nabla$ = [$\nabla_{x} ;\nabla_{y} ;\nabla_{z}$] is the 3D spatial gradient operator and α is a regularization weight that controls the amount of TV constraint applied. *M* is either chosen as a spatially varying weight that is estimated from the magnitude image to account for the nonuniform phase noise^12,27^, or a binary mask^12,17^, and $W = \left[ {W_{{\nabla_{x} mag}} ;W_{{\nabla_{y} mag}} ;W_{{\nabla_{z} mag}} } \right]$ is either the identity matrix I or a binary edge mask derived from the magnitude data^27^.

### Plug-and-Play ADMM formulation for QSM with non-local patch-based filters

We introduce a surrogate variable $v$ and enforce the variable substitution $\chi = v$ using ADMM^23^, rewriting Eq. (4) as

6 $$\frac{\mu }{2} ||M(F^{ - 1} DF\chi^{{}} - \phi )||_{2}^{2} + \alpha \varphi \left( v \right)^{{}} + \frac{\beta }{2}\left| {\left| {\chi - v + u} \right|} \right|_{2}^{2}$$

Here* u* is a scaled Lagrange multiplier^6^, $\mu$, $\alpha$ and $\beta$ are tunable weights. The minimization of Eq. (6) leads to the sequence of sub-problems

7 $$\chi^{n + 1} = {}_{min \chi }^{{}} \frac{\beta }{2}\left| {\left| {\chi - v + u} \right|} \right|_{2}^{2} + \frac{\mu }{2} ||M(F^{ - 1} DF\chi^{k} - \phi^{{}} )||_{2}^{{2}{}}$$

8 $$v^{n + 1} =_{\min v } \frac{\beta }{2}\left| {\left| {\chi - v + u} \right|} \right|_{2}^{2} + \alpha \varphi \left( v \right)$$

9 $$u^{n + 1} = u^{n} + \left( {\chi^{n + 1} - v^{n + 1} } \right)$$

Here Eq. (7) is a least squares inversion problem involving the forward QSM model, which promotes data consistency. With the intuition that Eq. (8), which involves the prior $\varphi \left( v \right)$, can be viewed as a denoising of “noisy” image ($\chi + u$), Venkatakrishnan et al.^20^ developed a variant of ADMM framework by suggesting that Eq. (8) can be replaced by a denoising algorithm $(D_{\sigma } )$. Since different applications may require different denoising properties, this plug-and-play framework allows us to easily use the most appropriate denoising algorithm. For the QSM model, this leads to the sequence of sub -problems

10 $$\chi^{n + 1} = {}_{min \chi }^{{}} \frac{\alpha }{2}\left| {\left| {\chi - v + u} \right|} \right|_{2}^{2} + \frac{\mu }{2} ||M(F^{ - 1} DF\chi^{k} - \phi^{{}} )||_{2}^{{2}{}}$$

11 $$v^{n + 1} = {}_{min v}^{{}} D_{\sigma } ( \chi + u)$$

12 $$u^{n + 1} = u^{n} + \left( {\chi^{n + 1} - v^{n + 1} } \right)$$

The ADMM based variable splitting converts the QSM reconstruction formulation into two decoupled reconstruction modules; one for Eq. (10), which promotes data consistency and one for Eq. (11), which can be interpreted as a subroutine for denoising. The major advantage of this modular structure of PnP-ADMM approach for QSM is that it allows denoising algorithms that are often not explicitly formulated as optimization problems to be used for QSM reconstruction. Additionally, this modular structure allows us to “plug-in” any suitable denoiser in Eq. (11). For our QSM reconstructions we use the BM4D^28^ as the denoiser, which is an extension of BM3D for volumetric data. The steps in the PnP-ADMM algorithm for QSM are shown in Supporting Information Fig. S1.

To further improve the quality and sharpness of QSM reconstructions, we include the “adding-noise-back” step^29–31^, which updates the local field based on the residual difference between the estimated image and local field. The adding-noise-back step has been shown to improve image quality for application such as denoising^32^, deblurring^32^, reconstruction of undersampled MRI images^31,33,34^, and more recently for QSM^17^.

The proposed method (Eqs. 6–12) has a fundamentally different purpose compared to previous approaches that use variable substitution. Previous approaches^12,15^ sought to overcome difficulties in using traditional optimization technique for regularized QSM and developed variable splitting based approached to reduce the reconstruction time. In^12^ (Eqs. 8–12), a variable substitution based approach was developed for L1 regularized QSM formulation to overcome the limitations of using non-linear conjugate gradient solver^12^. The variable substitution was applied to the data sparsity term and the resulting formulation allowed for rapid convergence of TV based QSM reconstructions. Similarly, in Ref^15^ (Eqs. 2–10), the variable splitting based approach was intended to accelerate the non-linear QSM formulation^35^, which is computationally slow to minimize with traditional optimization techniques. Using variable substitution, rapid minimization approaches were developed for non-linear QSM formulations that use TV or TGV as constraints. The existing variable splitting approaches are intended only to speed up the reconstruction time of QSM models that use explicit regularizers and they cannot be directly used for non-local patch-based denoisers such as BM3D, which do not have an explicit regularizer.

The primary goal of the PnP-ADMM based approach described here was to develop a framework that would allow for the use of state-of-the-art non-local patch-based denoising approaches that are not explicitly formulated as optimization problems for QSM reconstruction. In addition to analyzing the use of BM4D for QSM, we test the feasibility of using other non-local patch-based denoisers such as texture variation adaptive image denoising with nonlocal PCA (ACVA)^36^ and 2D slice-by-slice denoising using BM3D^19^ in the same PnP-ADMM reconstruction framework.

## Methods

### 2016 QSM challenge data

To validate the accuracy of single-orientation QSM reconstruction techniques with multi-orientation reconstruction, MRI data was obtained from the 2016 QSM reconstruction challenge^9^. The subject who provided data for the challenge gave informed consent to participate in a protocol approved by the Massachusetts General Hospital^9^. A single-orientation dataset and two multi-orientation reference standards, susceptibility tensor imaging (STI)^9^ data and Calculation Of Susceptibility through Multiple Orientation Sampling (COSMOS)^9^, were provided to participants to test and validate their reconstructions. The 3D gradient echo (GRE) data was acquired on a 3 T scanner (Tim Trio Model; Siemens Healthcare; Erlangen, Germany) equipped with a 32-channel head coil. Images were obtained in 12 different directions with respect to B0 using a 15-fold accelerated wave-CAIPI acquisition. Acquisition parameters were 1.06 × 1.06 × 1.06 mm^3^ isotropic resolution, 240 × 196 × 120 matrix size, flip angle = 15°, TE/TR = 25/35 ms, and bandwidth = 100 Hz/pixel.

### Brain MRI in glioblastoma

We obtained QSM data in 10 patients with GBM, who gave informed consent to participate under a protocol approved by the Institutional Review Board. Single echo gradient echo images were obtained in the axial orientation. Imaging was obtained on a 3 T scanner (Trio, Siemens Healthcare; Erlangen, Germany) using the following imaging parameters: spatial resolution = 0.86 × 0.86 mm^2^, slice thickness = 3 mm, acquired matrix size = 256 × 256 × 24, flip angle = 20°, TE/TR = 18/55 ms, and bandwidth = 444 Hz/pixel. The data was acquired using a 12-channel head coil.

Post-contrast T1 MPRAGE images were obtained with the following imaging parameters: spatial resolution = 0.98 × 0.98 mm^2^, slice thickness = 1 mm, acquired matrix size = 256 × 192 × 192, TE/TR/TI = 3.11/1750/950 ms, FA = 15° and bandwidth = 150 Hz/pixel) and FLAIR (spatial resolution = 0.938 × 0.938 mm^2^, slice thickness = 3 mm, matrix = 256 × 192 × 60, TE/TR/TI = 141/9420/2500 ms, FA = 170° and bandwidth = 287 Hz/pixel) were acquired after intravenous administration of a gadolinium-based contrast agent (MultiHance 0.1 mmol/kg, double dose).

### QSM reconstruction

We compared the results from the proposed patch-based reconstruction approach with Thikhonov regularized QSM formulation (Eq. (4) with the Thikhonov constraint used as $\varphi \left( \chi \right)$, called L2 in results), TV regularized QSM (Eq. (5) with W = I, called QSM-TV in results), morphology enabled dipole inversion^8^ (MEDI), and a non-linear QSM formulation that uses TGV^15^ as constraint (FANSI-TGV).

The FSL brain extraction tool^37^ was used to extract a brain mask from the magnitude image. The phase images were unwrapped using the Laplacian technique^12^, and transmit phase was removed by fitting and subtracting a fourth-order 3D polynomial^9^. The Laplacian boundary value method^12,38^ was used for background field removal. The different reconstructions were tested on the same local field. The reconstruction method was implemented Matlab (Natick, MA; Ver. R2020b) and tested on a computer with an Intel i5-9400 CPU with a processor frequency of 2.90 GHz, 6 cores and a total memory of 64 GB.The parameters for the reconstruction were empirically tuned to provide good visual image quality.

### Image analysis and statistics

Image quality was compared using visual analysis and also using global quality metrics such as root-mean-squared error (RMSE)^9^, structural similarity index metric (SSIM)^1,39^, high frequency error norm (HFEN)^9,40^, cross correlation (CC), blur metric^41^ and mutual information (MI)^9,42^. Manual segmentation was performed on the T1-weighted images using a combination of ITK-SNAP^43^ and FSL^44^ to segment the different anatomical brain regions. Mean susceptibility from ROIs were computed and compared using linear regression and Bland–Altman plots. The results of linear regression are reported as slope (β) and coefficient of determination (R^2^). The mean susceptibilities were further analyzed using Bland–Altman plots and bias was reported as mean bias ± 2σ. The statistical analysis was performed in Matlab (Natick, MA; Ver. R2020b).

To quantify the quality of the QSM reconstructions performed on GBM data, the reconstructions were assessed by three board-certified neuroradiologists (J.W.S, R.M.K, J.B.W). The assessment was done to quantify the overall image quality (IQ) and image sharpness (IS); performed as both a^1^ numerical grading of individual images on a scale 1–3 (1 = low, 3 = high, and half scores allowed) and^2^ numerical ranking of reconstructions on a scale 1–4 (4 = best, and 1 = worst) by doing a side-by-side comparison of IQ and IS. Four datasets were randomly chosen for training the three observers and the numerical grading and numerical ranking were performed on the remaining six GBM datasets. Scores provide by a fourth neuroradiologist (S.A.N) on the four training datasets were used as a reference to train three image quality raters. A detailed description of the image quality assessment criterion used for numerical grading is provided in Supporting Information Table S1^45^.

The assessment of numerical grading and numerical ranking were performed as separate sessions on two different days. The neuroradiologist viewed and ranked the images independently. They were blinded to the reconstruction type and the datasets were presented in a randomized order. The scores are reported as mean and standard deviation (mean ± SD). Images where the expert observers could easily track vessels, delineate anatomical features and easily identify the tumor vasculature and intratumoral hemorrhage received a higher grade. For the numerical grading of the 24 reconstructions (four reconstructions per datasets and total of six datasets), the observers were shown one reconstruction at a time. The observers were blinded to the reconstruction type and the order in which the image were shown was randomized. For the numerical ranking, the reconstructed images from the four techniques were viewed simultaneously by the neuroradiologists and the order of the four reconstructions were randomly shuffled. The numerical ranking was a measure of the comparative differences in image quality between the different reconstruction techniques. A Friedman test was used to verify the presence of a significant difference (level of significance at *p* < 0.05) in the median scores between at least two reconstruction methods. A post-hoc Conover multiple comparison test was performed with Bonferroni P-value adjustment to correct for the familywise error rate introduced when doing multiple comparisons.

## Results

### QSM reconstruction challenge

**Figure 1:**
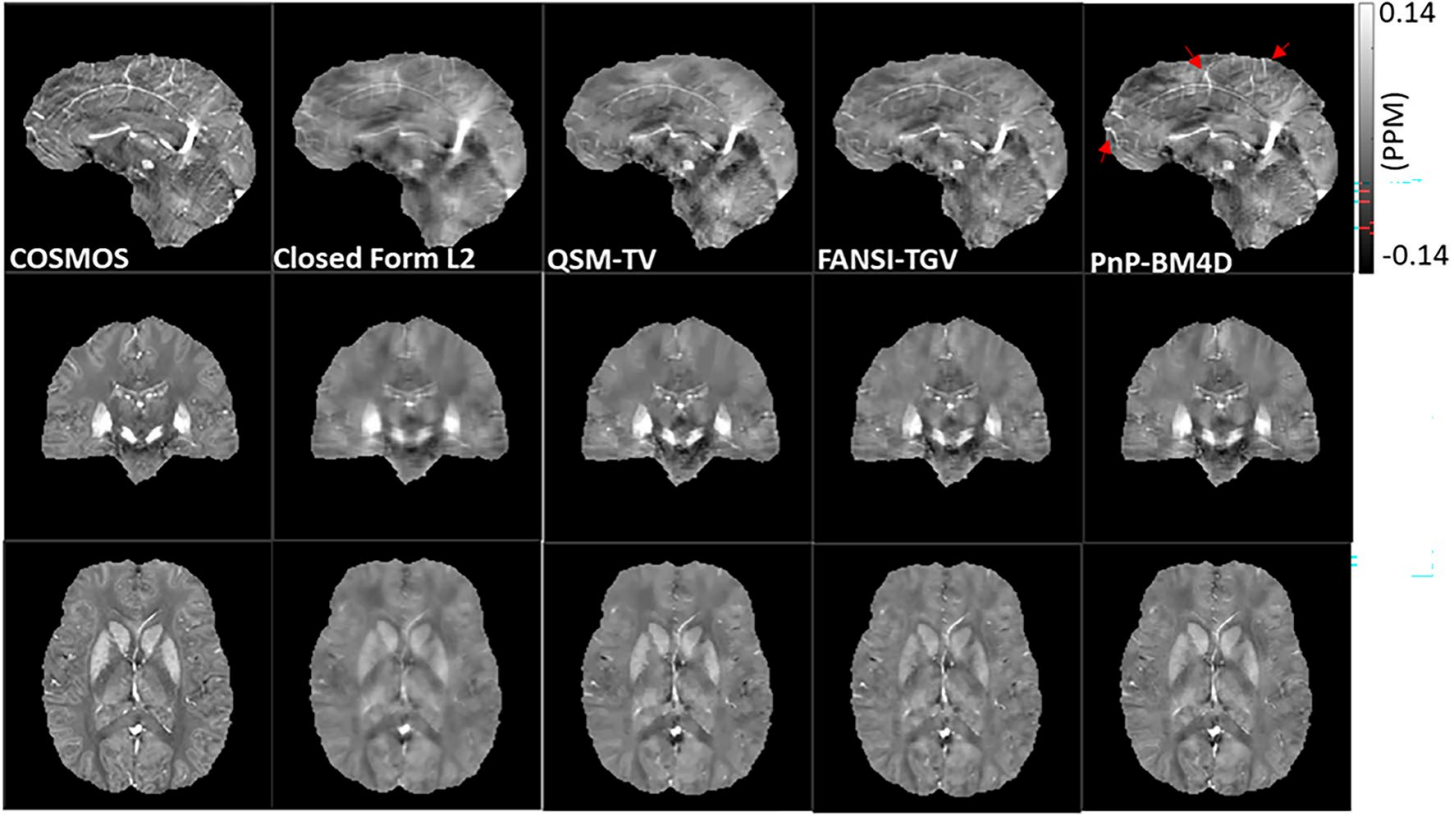
Reconstruction of susceptibility maps on the 2016 QSM reconstruction challenge dataset and comparison with the multi-orientation COSMOS. The red arrows show locations where the PnP-BM4D reconstructions exhibit sharper edges and improved vessel delineation.

For each of the four image reconstructions, we compared images with multi-orientation COSMOS using several global error quality metrics. Figure 1 shows a comparison of reconstructions using closed form L2, QSM-TV, FANSI-TGV and PnP-BM4D reconstruction formulations. RMSE was lowest in FANSI-TGV, followed by PnP-BM4D, QSM-TV and L2 (RMSE_FANSI-TGV_ = 60.8, RMSE_PnP-BM4D_ = 63.4, RMSE_QSM-TV_ = 64.8, RMSE_L2_ = 65.6). HFEN was lowest in PnP-BM4D, followed by FANSI-TGV, QSM-TV and L2 (HFEN_PnP-BM4D_ = 58.5, HFEN_FANSI-TGV_ = 59.9, HFEN_QSM-TV_ = 61.0, HFEN_L2_ = 63.8). SSIM was highest in PnP-BM4D, followed by FANSI-TGV, QSM-TV and L2 (SSIM_PnP-BM4D_ = 0.860, SSIM_FANSI-TGV_ = 0.851, SSIM_QSM-TV_ = 0.845, SSIM_L2_ = 0.825). CC was highest in PnP-BM4D, followed by FANSI-TGV, QSM-TV and L2 (CC_PnP-BM4D_ = 0.804, CC_FANSI-TGV_ = 0.796, CC_QSM-TV_ = 0.783, CC_L2_ = 0.758). MI was highest in PnP-BM4D, followed by L2, QSM-TV and FANSI-TGV (MI_PnP-BM4D_ = 0.475, MI_L2_ = 0.474, MI_QSM-TV_ = 0.462, MI_FANSI-TGV_ = 0.461). The global image quality metrics are reported in Table 1. There was good correlation between the standard COSMOS reconstruction and QSM-TV (β = 0.86, R^2^ = 0.98, *p* < 0.05), FANSI-TGV (β = 0.76, R^2^ = 0.98, *p* < 0.05), and PnP-BM4D (β = 0.84, R^2^ = 0.98, *p* < 0.05). There was no significant bias detected in the reconstructions using PnP-BM4D (bias = 0.007 ± 0.012), FANSI-TGV (bias = 0.007 ± 0.014) and QSM-TV (bias = 0.004 ± 0.011). There was improved visual conspicuity of finer anatomical details, such as small caliber vessels, with PnP-BM4D as compared to the other reconstruction techniques (location shown by red arrows in Fig. 1). This improved visual conspicuity was quantitatively reflected by the lower HFEN, and higher SSIM.

**Table 1:** Comparison of global error quality metrics.

	L2	FANSI-TGV	QSM-TV	PnP-BM4D
RMSE	65.62	60.8	64.76	63.42
HFEN	63.83	59.99	61.04	58.5
SSIM	0.825	0.850	0.845	0.860
MI	0.474	0.461	0.462	0.475
CC	0.758	0.796	0.783	0.804

Mean root mean squared error (RMSE), high frequency error norm (HFEN), structural similarity index (SSIM) and mutual information (MI), and correlation coefficient (CC) are reported for L2, FANSI-TGV, QSM-TV and PnP-BM4D reconstructions.

### PnP-ADMM QSM in patients diagnosed with Glioblastoma

**Figure 2:**
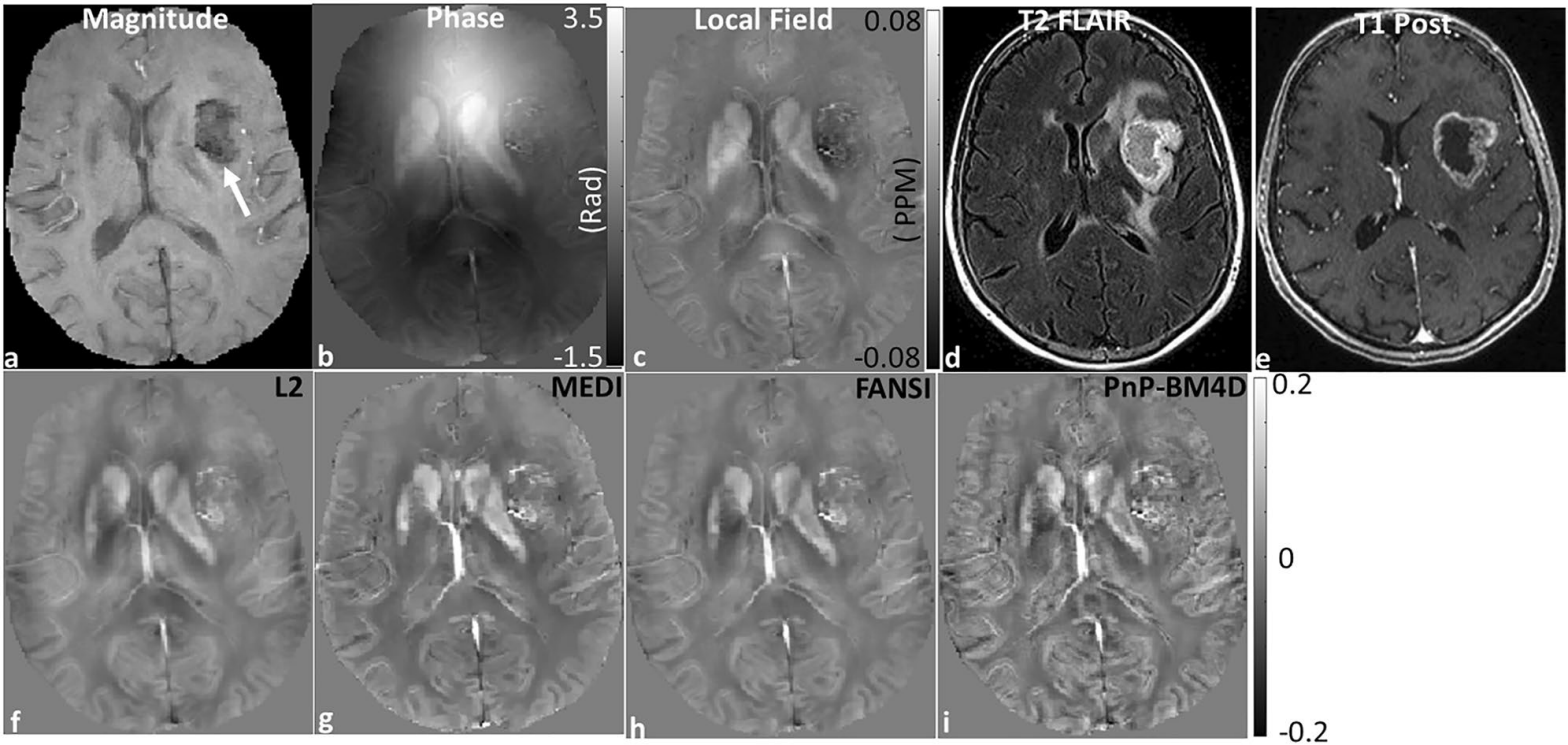
Reconstruction of susceptibility maps from a patient with GBM. (**a**) The glioblastoma appears hypointense in the T2*-weighted magnitude image (white arrow). The corresponding (**b**) phase image, (**c**) local field map, (**d**) T2 FLAIR and (**e**) T1 Post contrast image are displayed. QSM reconstructions from (**f**) closed form L2, (**g**) MEDI, (**h**) FANSI, and (**i**) PnP-BM4D are shown. Tumor features are more conspicuous and show less blurring of vascularity in the PnP-BM4D reconstructions. Additionally, there is less blurring and higher contrast in grey matter cortical regions.

**Figure 3:**
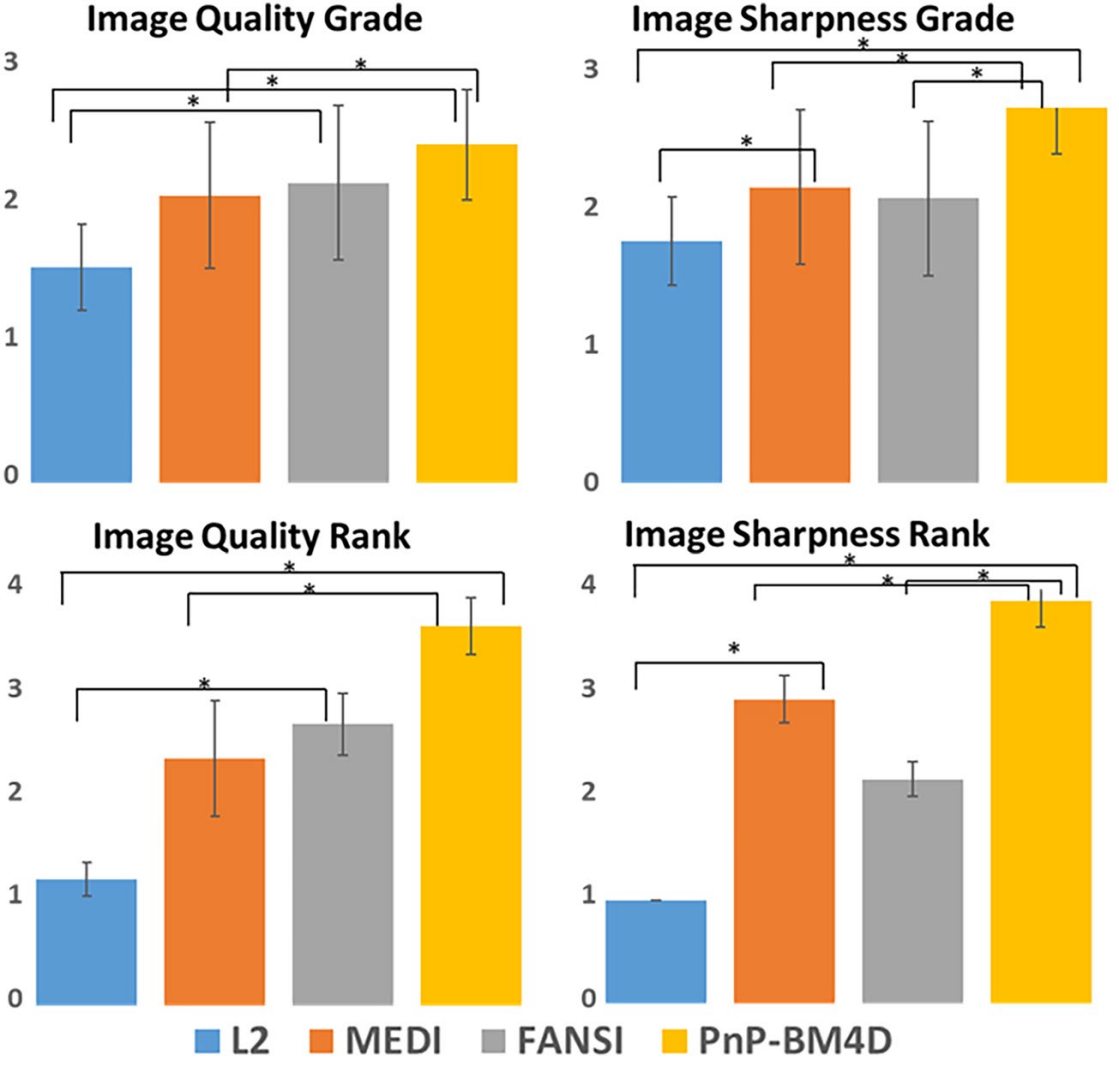
Results from the numerical grading and numerical ranking of QSM reconstructions from 6 patients diagnosed with GBM. The average scores for overall image quality (IQ) and image sharpness (IS) are shown. An asterisk is used to indicate pairs that have a significant (P < 0.05) difference in the median scores.

The results from the GBM data are shown in Fig. 2.The reconstruction time for L2 was ~ 0.13 s, FANSI was ~ 1.8 min, MEDI was ~ 6.1 min and BM4D was ~ 9.4 min. The location of the tumor is shown by an arrow in Fig. 2a. The reconstructions from Thikhonov regularized QSM (L2, Fig. 2f) show blurring of structures (blur = 0.35) as compared to other MEDI (Fig. 2g, blur = 0.27), FANSI (Fig. 2h, blur = 0.27), and BM4D (Fig. 2i, blur = 0.24) reconstructions. The TV based reconstructions show good artifact removal, but with texture degradation and blurring of fine structures (Fig. 2g,h). The reconstructions from the BM4D based non-local denoising approach have high quality and structures such as edges in the gray-white matter boundaries and vessels appear sharper. PnP-BM4D reconstructions (IQ_Grade_ = 2.4 ± 0.4, IS_Grade_ = 2.7 ± 0.3, IQ_Rank_ = 3.7 ± 0.3, IS_Rank_ = 3.9 ± 0.3) consistently scored higher than MEDI (IQ_Grade_ = 2.1 ± 0.5, IS_Grade_ = 2.1 ± 0.6, IQ_Rank_ = 2.4 ± 0.6, IS_Rank_ = 2.9 ± 0.2) and FANSI-TGV (IQ_Grade_ = 2.2 ± 0.6, IS_Grade_ = 2.1 ± 0.6, IQ_Rank_ = 2.7 ± 0.3, IS_Rank_ = 2.2 ± 0.2) reconstructions. The mean IQ and IS scores for grading and ranking are reported in Tables 2 and 3 respectively. Pairs with a statistically significant difference in the medians are shown in Fig. 3. We found a statistically significant difference in IS (for grade and rank) between PnP-BM4D and the other three reconstruction techniques. The results of the multiple comparisons testing are reported in Supporting Information Tables S2 and S3.

**Table 2:** Mean image quality (IQ) grade and image sharpness (IS) grade from 6 GBM datasets (Scale 1–3; 1 = least, 3 = most).

	L2	MEDI	FANSI	PnP-BM4D	*p*
Image quality grade	1.6 ± 0.3	2.1 ± 0.5	2.2 ± 0.6	2.4 ± 0.4	*p* < 0.05
Image Sharpness grade	2 ± 0.4	2 ± 0.5	1.8 ± 0.4	2.6 ± 0.3	*p* < 0.05

Statistical testing was performed using Friedman chi-squared test. Test for multiple comparisons using Conover’s test is reported in Supporting Information Table S2. PnP-BM4D scored the highest when compared to L2, MEDI and FANSI reconstructions.

**Table 3:** Mean image quality (IQ) rank and image sharpness (IS) rank from 6 GBM datasets (Scale 1–4; 4 = best, 1 = worst).

	L2	MEDI	FANSI	PnP-BM4D	*p*
Image quality Rank	1.2 ± 0.2	2.4 ± 0.6	2.7 ± 0.3	3.7 ± 0.3	*p* < 0.05
Image Sharpness Rank	1 ± 0	2.9 ± 0.2	2.2 ± 0.2	3.9 ± 0.3	*p* < 0.05

Statistical testing was performed using Friedman chi-squared test. Test for multiple comparisons using Conover’s test is reported in Supporting Information Table S3. PnP-BM4D scored the highest when compared to L2, MEDI and FANSI reconstructions.

**Figure 4:**
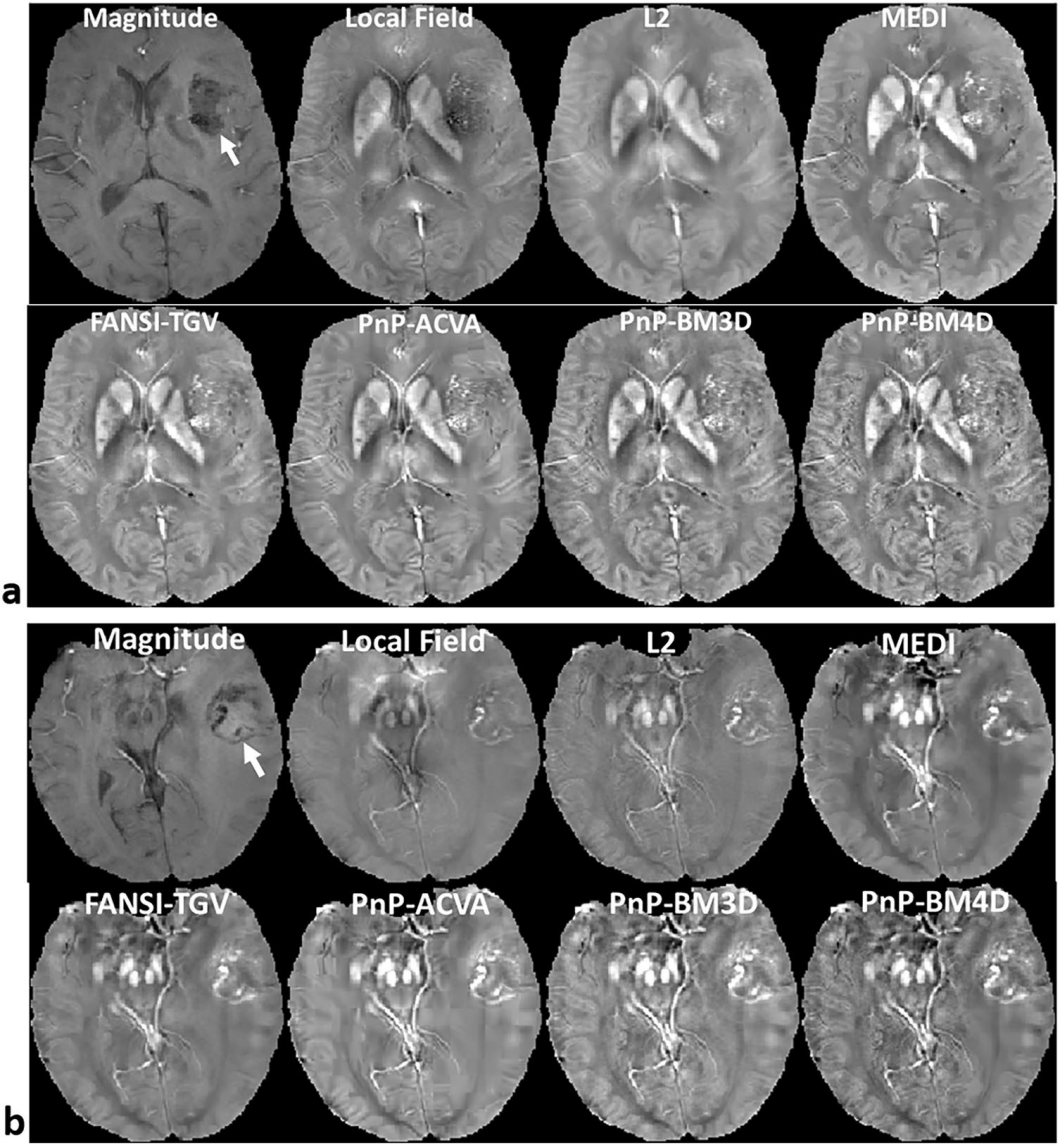
Two examples showing the “plug-and-play” modular nature of PnP-ADMM reconstruction algorithm. Reconstructions from 3 different denoisers, namely ACVA, BM3D and BM4D with GBM. The location of the tumor is indicated by the arrow in the magnitude image. The non-local patch-based denoisers perform well at preserving features such as bright vessels and fine textures, especially in the grey matter cortical regions.

We tested the modular nature of the proposed PnP-ADMM QSM formulation by using two other non-local patch-based denoisers, namely ACVA and BM3D. The results are shown in Fig. 4. The two other non-local patch-based denoisers also show good visual sharpness, vessel conspicuity and preserve contrast in the grey matter cortical regions.

## Discussion

We developed and investigated the performance of an iterative reconstruction algorithm for QSM that integrates non-local patch-based denoising to improve the quality of magnetic susceptibility maps. There are several important findings to our work. We developed a framework to apply PnP priors to iterative QSM reconstruction, enabling the flexible use of several different types of denoisers that were previously inaccessible to QSM or potentially would have required the development of a new algorithm for each. The motivation for the use of these denoisers is the structure of underlying clinical data. Brain magnetic susceptibility images show many regions of high structural similarity including small vessels, cortical structures, and left-to-right symmetry. These structurally similar regions may be far apart in the image domain, appearing in different regions with dissimilar structures in between. We surmised that non-local patch-based denoising approaches would have distinct advantages compared to local regularizers used in QSM reconstruction, since they can more effectively leverage these similarities to remove reconstruction artifacts. We developed for the first time a non-local patch-based QSM reconstruction and show that it achieves high image quality magnetic susceptibility maps of human brain.

Our work demonstrates how non-local patch-based denoising techniques can be integrated with QSM reconstruction using PnP priors, which is a method that was adapted from the field of image denoising^20^. PnP is powerful because of its flexibility; the user can choose an available or novel non-local patch-based denoiser and apply it to the problem of QSM reconstruction. Traditionally, non-local patch-based denoisers could not be used in a regularized reconstruction framework. In addition, depending on the type of regularization constraint used, the reconstruction framework often required modifications of the underlying optimization algorithm, requiring additional expertise and validation. PnP is flexible in that non-local patch-based denoisers can be tested or used without revision of the optimization algorithm. Suitable patch-based denoisers can be chosen based on the specific QSM application and the same reconstruction framework can be used to test the different denoisers. This property of the PnP framework is highly beneficial because different QSM applications may need different denoising approaches. For example, the type of features and textures seen in neuroimaging applications might be different from those seen in QSM of the liver^46^. Hence the two applications may need different regularizers or denoisers. While the analysis in this paper is limited to neuroimaging applications, the framework described here can be easily be applied to other QSM applications.

Several ADMM based approaches^12,15^ were developed for regularized QSM. The aim of these techniques was to accelerate the convergence of the cost functional as it cannot be rapidly achieved with traditional gradient descent-based formulations. Rapid variable substitution-based approaches were developed for L2 and L1 regularized linear QSM models^12^ and non-linear models^15^. These approaches were formulated for QSM models that use fixed regularizers like TV or TGV and enforce image models such as piecewise-smoothness or piecewise-constant constraints. For applications such as neuroimaging, these models may not conform optimally with the underlying image as it frequently contains several fine features and textures. While these models produce images with low mean-squared error and high ROI accuracy, the removal of artifacts is achieved with loss of contrast, blurring of fine structures such as vessels, and smoothing of features such as edges in low contrast regions. Patch-based denoising approaches on the other hand are more data driven and do not enforce any smoothness constraints on the image. The removal of artifacts is performed by combining similar patches and filtering the them in a transformation domain. Hence patch-based approaches may be more suited for neuroimaging applications. These patch-based techniques are more data driven and better suited to handle images with sharp edges, fine features and textures. Recently, several deep learning based approaches have been developed for QSM reconstruction that aim to leverage the improved image quality using patch-based QSM reconstructions. In QSMnet a modified 3D U-net was used to reconstruct QSM images from single orientation data and 3D patches from multi-orientation COSMOS maps were used as ground truths for the QSM maps^47^. Since acquiring COSMOS can be a challenge, the DeepQSM approach developed a patch-based training model using synthetic data^48^. In DeepQSM, a fully convolutional deep neural network was trained to learn the physics based dipole convolution forward model and directly invert the dipole convolution during reconstruction. There have also been hybrid approaches that have been developed recently to use deep learning models as denoisers in an iterative reconstruction framework^49,50^. An advantage that the hybrid approaches have over the purely deep learning based methods is that the hybrid approaches have the ability to include physics based models in the reconstruction pipeline, thereby achieving better image quality and ensuring higher fidelity to the acquired data. A ADMM-based variable splitting approach was also included in to accelerate the iterative reconstruction.

The 2016 QSM reconstruction challenge^9^ data provided a platform to compare reconstruction from single orientation acquisitions with multi-orientation gold standard. Image quality was assessed using image quality metrics such as SSIM, RMSE, and HFEN. Many of the best scoring approaches used sparsity-enforcing constraints. While the analysis of the QSM maps from techniques that scored the highest in each category showed similar error metrics, the visual appearance of these images were substantially different^9^. It was found that optimizing the weights of the QSM algorithms to minimize error metrics led to over-smoothing of images and loss of sharpness of fine features such as vessels^9^. In addition, approaches that performed well in one image quality category performed poorly in another. Recent analysis^10^ suggested that several factors such as incomplete background field removal, anisotropic susceptibility, flow, motion artifacts, improper co-registration, noise, and microstructure contributions may be possible sources of inconsistencies between the multi-orientation ground truth images and the single orientation phase data. Despite these limitations, the 2016 challenge data provides a valuable platform to test the performance of new reconstruction approaches and verify their efficacy.

High quality reconstructions were estimated using the proposed patch-based QSM reconstruction formulations. We found that for the images reconstructed using the 2016 QSM challenge data, lower HFEN and higher SSIM and higher MI were estimated using PnP-Bm4D when compared with QSM-TV, and FANSI-TGV reconstructions. While improvement of error metrics such as RMSE is achieved at the cost of blurring of edges and smoothing of features, PnP-BM4D was able to achieve a good balance between reduction of artifacts and preservation of fine features. There was good agreement in the mean susceptibility estimated by PnP-BM4D, FANSI-TGV and QSM-TV as compared to COSMOS and we were unable to detect a significant bias. Features such as bright veins that were present in the COSMOS images were better visualized in PnP-BM4D reconstructions while some smoothing was visible in reconstructions using QSM-TV and FANSI-TGV reconstruction. Reconstruction of QSM image with sharp vessels could be useful in applications such as automated vein segmentation^51^ or venous oximetry^52^.

Several recent studies have investigated magnetic susceptibility in the tumoral microenvironment^53,54^. Due to their short T2* relaxation, hemorrhagic lesions and diamagnetic calcifications in brain tumors may show similar appearance on gradient echo images^55^. Since QSM can separate similarly short T2* signals by their paramagnetic or diamagnetic shift, it can differentiate these lesions and provide additional value to conventional T2 FLAIR and T1 post-contrast images. These lesions and other tumor vascular components are often small compared to the tumor size, so the development of QSM methods that preserve these fine features are important. Moreover, smoothing that is caused due to the use of piecewise-smoothness or piecewise-constant constraints are avoided using PnP-BM4D. PnP-BM4D reconstructions on the GBM data had consistently better image quality compared to L2, MEDI and FANSI, and this is reflected in the higher grade and higher rank for IQ and IS assessment. There was a statistically significant difference in IS grade and IS rank between PnP-4D and the three other reconstruction techniques.

In addition to BM4D, we tested the PnP modular nature of the proposed algorithm using two other patch-based denoising approaches, ACVA and BM3D, without changing the reconstruction algorithm and by just replacing the denoisers in Eq. (11). The BM3D algorithm is a block-matching and collaborative filtering approach designed to denoise 2D images. Similar 2D patches of the image are grouped in a larger 3D array and the coefficients are filtered after the application of a suitable 3D transformation. ACVA uses a combination of adaptive clustering of patches with variation adaptive filtering in the PCA domain. The results on the GBM dataset showed that the non-local patch-based denoisers produced images with high contrast, especially in the gray matter cortical regions and sharpness of the vessels in well preserved.

## Conclusion

We developed a PnP-ADMM based framework for QSM reconstructions which allows for the use of patch-based denoising to achieve high quality reconstructions without the loss of edge sharpness and blurring of features in the image. The proposed technique does not depend on the use of spatial priors to reduce the blurring of edges due to data regularization or the estimation of spatially varying phase noise for the data fidelity term. The proposed reconstruction formulation performs better than existing popular QSM approaches when compared using global quality metrics such as HFEN, SSIM, CC and MI, and also achieves sharper edges and better retention of fine features in the reconstructed images.

## Supplementary Information

Supplementary Information.

## Data Availability

The datasets used and/or analyzed during the current study are available from the corresponding author on reasonable request.
