# Supplementary material for: Quantitative susceptibility mapping using plug-and-play alternating direction method of multipliers
Source: Sci Rep. 2022 Dec 15;12:21679. doi: 10.1038/s41598-022-22778-w (PMC9755132; doi:10.1038/s41598-022-22778-w)
Supplement: Supplementary file 1 — Supplementary Information. [file 41598_2022_22778_MOESM1_ESM.pdf]

Supporting Information

For m=1:N2

For n=1:N1

$$\chi^{n+1} \ =_{min\,\chi} \frac{\alpha}{2}||\chi - v + u||_2^2 + \frac{\mu}{2}\ ||M(F^{-1}DF\chi^n \ - \phi^m)||_2^2$$

$$v^{n+1} \ =_{min\,v} \frac{\beta}{2}||\chi - v + u||_2^2 + \alpha\varphi(v)$$

$$u^{n+1} = u^n + (\chi^{n+1} - v^{n+1})$$

End

$$\phi^{m+1} = \phi^m + (\phi - F^{-1}DF\chi^k).$$

End

Supporting Information Figure S1: Algorithm of the proposed PnP-ADMM algorithm for QSM. The iterative update of ϕ in the outer loop is the "adding noise back" step. Since the use of denoisers may cause smoothing of edges along with removal of noise and artifacts, the adding-noise-back step updates the local field and information about sharp features and edges are added back to the reconstruction.

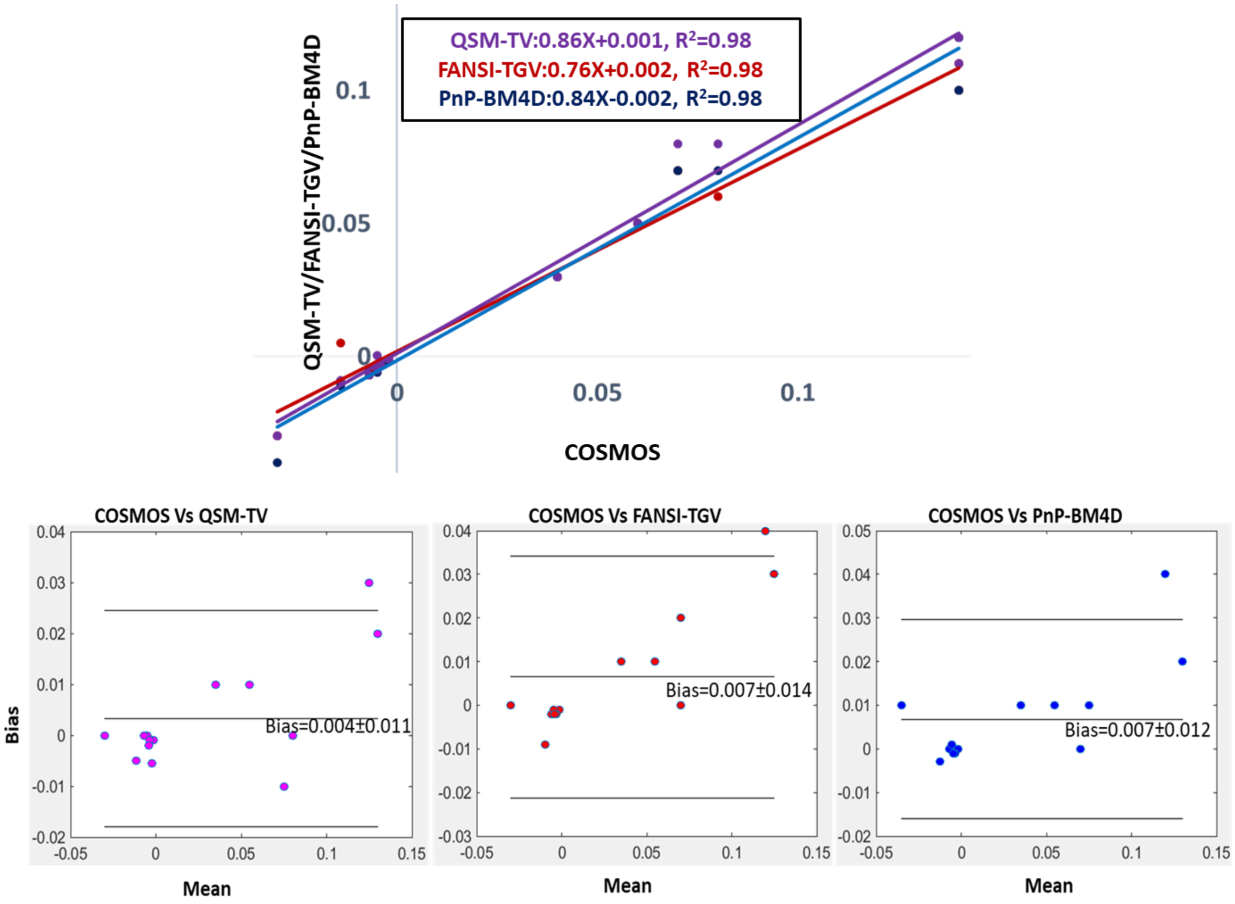

Supporting Information Figure S2. Results from the 2016 QSM reconstruction challenge dataset and comparison with the multi-orientation COSMOS. The mean magnetic susceptibilities estimated from PnP-BM4D, FANSI-TGV, and QSM-TV have good correlation with the multiorientation COSMOS.

Assessment of Image quality (IQ)	
Image Grade	Interpretations
1	Images are very blurry, with low contrast, and accurate delineation of anatomical features is not feasible
2	Images show some blur, have moderate contrast, and accurate delineation of anatomical features is feasible with some difficulty.
3	Images are sharp, with high contrast, and accurate delineation of anatomical features is easy.
**Assessment of Image sharpness (IS)**	
Image Grade	Interpretations
1	Vessels have significant blur, with low contrast, and accurate tracking of vasculature in the image is difficult. When present, significant blur is visible in the tumor vasculature, and differentiating between tumor vasculature, and tumor hemorrhage is difficult.
2	Vessels show some blur, have moderate contrast, and accurate delineation of vasculature is feasible in 50-80% of vessels. When present, some blur is visible in the tumor vasculature, and differentiating between tumor vasculature, and tumor hemorrhage is feasible with some difficulty.
3	Vessels are sharp, with high contrast, and accurate delineation of vasculature is feasible in more than 80% of vessels. When present, tumor vasculature is sharp and differentiating between tumor vasculature and tumor hemorrhage is easy.

Supporting Information Table S1: The scoring criteria used for the assessment of GBM data.

Image Quality Rank (P<0.05)			
	FANSI	L2	PnP-BM4D
L2	**0.00016**	-	-
PnP-BM4D	**0.0026**	**1.8e-7**	-
MEDI	1	**0.00087**	**0.00049**
**Image Sharpness Rank (P<0.05)**			
	FANSI	L2	PnP-BM4D
L2	**0.0025**	-	-
PnP-BM4D	**2e-6**	**7.4e-9**	-
MEDI	**0.0012**	**6.8e-7**	**0.0115**

Supporting Information Table S2: Results of the Friedman test and the post-hoc Conover multiple comparison test with Bonferroni p-value adjustment to adjust for the family-wise error rate introduced when doing multiple comparisons. The pairs with a statistically significant difference (P<0.05) in medians are highlighted in bold.

Image Quality Grade (P<0.05)			
	FANSI	L2	PnP-BM4D
L2	**0.01**	-	-
PnP-BM4D	0.063	**4e-5**	-
MEDI	.5535	0.3674	**0.0016**
**Image sharpness Grade (P<0.05)**			
	FANSI	L2	PnP-BM4D
L2	0.3489	-	-
PnP-BM4D	**0.0006**	**1.6e-7**	-
MEDI	1	**0.0229**	**0.009**

Supporting Information Table S3: Results of the Friedman test and the post-hoc Conover multiple comparison test with Bonferroni p-value adjustment to adjust for the family-wise error rate introduced when doing multiple comparisons. The pairs with a statistically significant difference (P<0.05) in medians are highlighted in bold.

**Metric**	**Description**	**Reference for additional details**
High Frequency error norm (HFEN)	$\xi_{HFEN} = \frac{\| LoG(\chi) - LoG(\chi_{ref}) \|_2}{\| LoG(\chi_{ref}) \|_2}$; where LoG is a rotationally symmetric Laplacian of Gaussian.	1,2
Root mean squared error (RMSE)	*RMSE*= 100 * norm(χ - χ_{ref}) / norm(χ_{ref})	2
Structural Similarity Index Metric (SSIM)	$\xi_{HFEN} = \sum_i \frac{(2\mu_{\chi,i} + C_1)(2\mu_{\chi_{ref},i} + C_2)}{(\mu^2_{\chi,i} + \mu^2_{\chi_{ref},i} + C_1)(\sigma^2_{\chi,i} + \sigma^2_{\chi_{ref},i} + C_1)}$	3
Correlation coefficient (CC)	*CC*=corrcoef(χ , χ_{ref}) in Matlab	
Blur metric	A reference image-free metric to quantify the amount of blur in the image. It is normalized to a range of 0–1, larger values signify more blurring in the image. The metric is computed by comparing the edge information of the original image and a low-pass filtered version of the image. A detailed explanation of the blur metric is provided in Ref 4.	4

Mutual Information (MI)	$MI(\chi, \chi_{ref}) = H(\chi) + H(\chi_{ref}) - H(\chi, \chi_{ref})$, where H is the entropy.	5

Supporting information Table S4: Different error metrics used to compare the QSM reconstructions.

Reference for error metrics:

1. S. Ravishankar and Y. Bresler, "MR image reconstruction from highly undersampled k-space data by dictionary learning," IEEE Transactions on Medical Imaging, vol. 30, no. 5, pp. 1028–1041, 2011.
2. C. Langkammer, F. Schweser, K. Shmueli , et al. "Quantitative susceptibility mapping: report from the 2016 reconstruction challenge". Magn Reson Med. 2018;79:1661-1673.
3. Z. Wang, A. C. Bovik, H. R. Sheikh, and E. P. Simoncelli, "Image quality assessment: from error visibility to structural similarity," IEEE Transactions on Image Processing, vol. 13, no. 4, pp. 600– 612, 2004.
4. F. Crete, T. Dolmiere T, P. Ladret, et al. "The blur effect: perception and estimation with a new no-reference perceptual blur metric". Proc. SPIE 6492, Human Vision and Electronic Imaging XII, 64920I (12 February 2007).
5. R. Moddemeijer R. "On Estimation of Entropy and Mutual Information of Continuous Distributions". Signal Processing. 1989;16:233–246
